# A novel transoral extraction technique in robotic resection of gastroduodenal tumors: a case series

**DOI:** 10.1007/s00423-026-04062-x

**Published:** 2026-04-28

**Authors:** Laleh Foroutani, Bahaa I. Aburayya, Andrew Gonzalez, Suwayda Ali, Amir Ashraf Ganjouei, Jaeyun Jane Wang, Kimberly Kirkwood, Carlos Corvera, Abdul Kouanda, Adnan Alseidi, Mohamed Abdelgadir Adam

**Affiliations:** 1 Department of Surgery, University of California, San Francisco, San Francisco, USA; 2 Faculty of Medicine, Jordan University of Science and Technology, Irbid, Jordan; 3 Division of Gastroenterology, Department of Medicine, University of California, San Francisco, San Francisco, CA USA

**Keywords:** Robotic-assisted surgery, Transoral specimen extraction, Gastroduodenal tumors, Natural orifice specimen extraction surgery (NOSES), Minimally invasive surgery, Endoscopic transoral approach

## Abstract

**Background:**

Robotic-assisted resection of non-adenocarcinoma gastroduodenal tumors offers equivalent oncologic safety while preserving the benefits of minimally invasive surgery. Transoral extraction augments these benefits by reducing the need for a larger extraction incision. This study explores the feasibility of endoscopic transoral extraction of robotically resected gastroduodenal tumors.

**Methods:**

Patients underwent robotic gastroduodenal tumor resection by an experienced robotic surgeon. Resected specimens were secured in an EndoCatch bag and retrieved transorally using an upper gastrointestinal endoscope.

**Results:**

Five patients (median age 46 years, 40% female) were included. Patients were ASA class II/III. Tumor sizes ranged from 2.1 to 9.0 cm, located at the gastroesophageal junction, gastric fundus, lesser curvature, and proximal duodenum. Pathological diagnoses included leiomyoma, gastrointestinal stromal tumor (GIST), and glomus tumor. Robotic resection utilized 8-mm abdominal ports. No intraoperative adverse events occurred, and all specimens were retrieved intact. The median console time was 94 min, and 80% of cases had estimated blood loss of less than 20 cc. The median hospital stay was two days. Most patients (80%) resumed clear liquid diet by postoperative day one and progressed to full-liquid diet by day two. No patients required narcotics for postoperative pain management, with 80% managed with oral acetaminophen alone. No esophageal or oropharyngeal complications, readmissions, mortality, or incisional hernias occurred during the follow-up.

**Conclusion:**

This case series demonstrates the technical feasibility of robotic-assisted resection of gastroduodenal tumors with structured transoral extraction using real-time endoscopic guidance, representing a technical extension of established NOSES principles in the robotic surgery setting. Prospective multicenter studies are needed to validate safety and broader applicability.

## Background

Robotic-assisted approaches have expanded the application of minimally invasive approaches in the management of gastroduodenal tumors by providing superior precision, enhanced visualization, and improved ergonomics compared to traditional approaches. This minimally invasive approach may offer potential benefits, such as reduced postoperative pain, shorter hospital stays, and quicker recovery times, making it particularly appealing for non-adenocarcinoma gastroduodenal tumors, which may require complex resections. Despite these advantages, specimen extraction necessitates a relatively larger abdominal incision, potentially minimizing some of the benefits of minimally invasive surgery by increasing postoperative pain, use of opioids, risk of wound complications, and recovery time.

Natural orifice specimen extraction surgery (NOSES) has emerged as an innovative technique to preserve the benefits of minimally invasive surgery by avoiding larger abdominal incisions for specimen extraction [1]. By utilizing natural body orifices, NOSES allows for specimen retrieval while minimizing abdominal wall trauma, with demonstrated benefits in postoperative complications and cosmetic outcomes [2]. While transvaginal and transrectal routes represent the most widely utilized NOSES approaches, transoral extraction has also been described in the laparoscopic setting [3].

Despite growing interest in this approach, its application in the robotic surgery setting remains unexplored. Transoral extraction could further enhance the minimally invasive nature of robotic surgery by eliminating the need for a relatively larger abdominal incision, reducing risks associated with traditional mini-laparotomy extraction, such as wound infections, incisional hernias, and postoperative pain [4, 5]. However, concerns remain regarding the safety and feasibility of this approach, particularly with respect to potential complications such as esophageal or oropharyngeal injury during specimen extraction [6].

Given the relatively novel application of transoral extraction in robotic surgery, it is critical to assess its practicality in clinical practice. Establishing the feasibility of this technique will be essential in determining its potential to become an option in robotic gastroduodenal surgery, particularly for patients who may benefit from less invasive options for tumor resection and specimen retrieval. This case series aimed to evaluate the feasibility of transoral specimen extraction following robotic-assisted resection of gastroduodenal tumors by examining patients with tumors of varying sizes and locations along the gastroduodenal tract. The goal was to determine whether transoral extraction offers a more minimally invasive alternative for specimen extraction.

## Methods

Adult patients (> 18 years) who presented for surgical evaluation at the University of California, San Francisco (UCSF) were considered for inclusion in this case series. Eligible patients had no history of prior gastroduodenal abdominal surgery and were classified as ASA II or III. All patients underwent preoperative esophagogastroduodenoscopy (EGD) with endoscopic ultrasonography of the gastrointestinal masses. Patients with esophageal varices or stenosis were excluded. CT scans were used for size determination. Selection was based on the smallest tumor dimension, which represents the limiting diameter for safe transesophageal extraction to reduce the risk of complications associated with transoral extraction [7]. Histologic and pathologic criteria were restricted to patients with non-adenocarcinoma gastric masses and no evidence of metastatic disease. All patients were informed about the risks and benefits of the procedure. Patient data were managed according to HIPAA guidelines and UCSF’s institutional regulations to ensure privacy and compliance with ethical standards.

### Surgical technique

All procedures were performed by a single surgeon with dual expertise in robotic surgical oncology and advanced endoscopy, in compliance with institutional ethical standards and the principles of the Helsinki Declaration [8]. The surgical team was led by the primary surgeon (M.A.A.), a board-certified and fellowship-trained surgical oncologist with extensive experience in robotic and minimally invasive surgery (MIS). The team included a bedside assistant, an anesthesiologist, a circulating nurse, and a surgical technologist. The procedure was performed using the da Vinci Xi Surgical System (Intuitive Surgical, Mountain View, CA, USA) as the robotic platform.

Following the acquisition of informed consent, the patient was brought to the operating room, and general anesthesia was administered. An endotracheal tube secured the airway, and an orogastric tube was placed. Preoperative prophylactic antibiotics and subcutaneous heparin were administered to minimize the risk of infection and thromboembolic events, respectively. The patient was positioned in a supine position, and the abdomen was prepped and draped in a sterile fashion. A Veress needle was used to insufflate the abdomen and establish access to the abdominal cavity. A minimum of three 8-mm robotic ports were placed at the level of the umbilicus, with the exchange of one of these ports for a 5-mm utility port infraumbilically when needed for added ease of use of laparoscopic instruments. Before docking the robot, meticulous care was taken to ensure optimal visualization of the anatomy, with any visible abnormalities recorded. All patients except one were positioned in the reverse Trendelenburg position, and their abdominal ports were docked accordingly. The da Vinci Xi robotic arms were adjusted and aligned with the target anatomy, ensuring no undue tension on the docked ports. The primary surgeon controlled the system from the console while a surgical trainee assisted at the bedside. One patient, who had extensive abdominal adhesions, underwent laparoscopic adhesiolysis prior to docking. Endoscopic guidance, with lesion marking, facilitated robotic identification and precise mapping of the tumor. The gastric masses were carefully dissected using various techniques, including wedge or endoluminal resection from the gastric wall, with particular attention to preserving critical anatomical landmarks such as the gastroesophageal junction (GEJ) or the vagus nerve. The resection was planned to ensure negative margin resection and performed using robotic monopolar scissors.

**Fig. 1:**
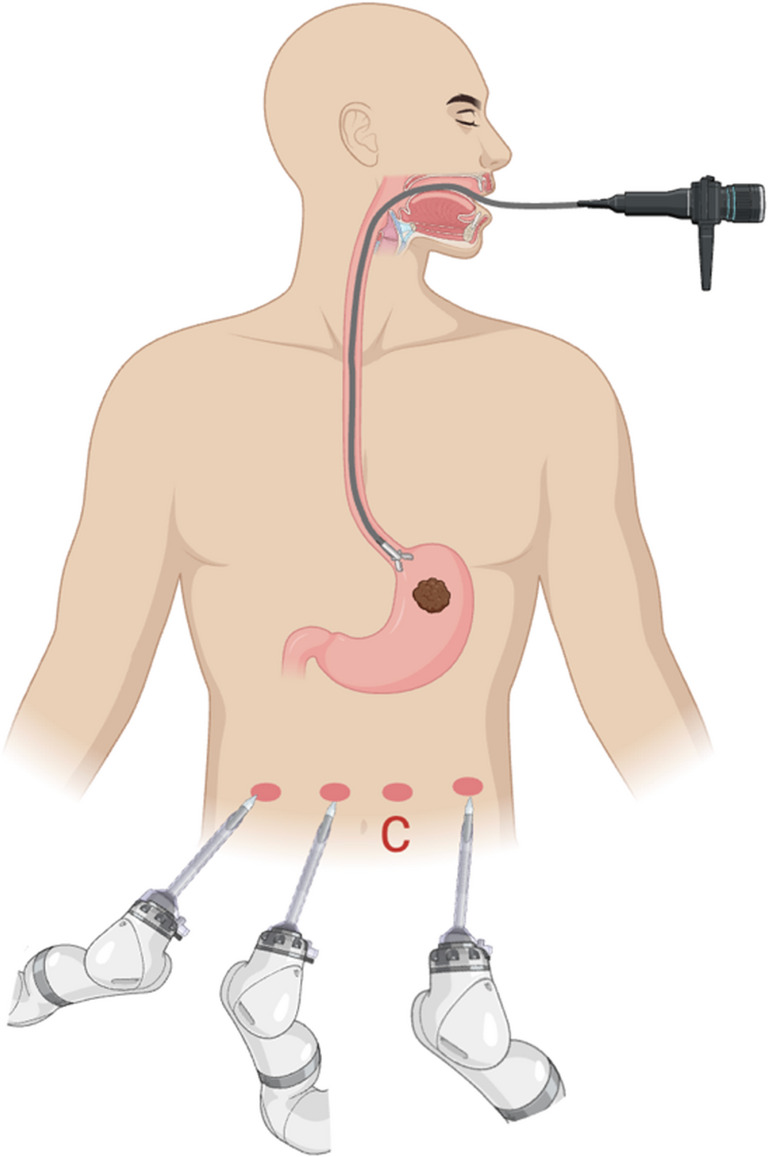
Illustration of the transoral extraction technique following robotic-assisted resection of a gastroduodenal tumor- created with BioRender.com

Following the complete resection of the lesions, a lubricated EndoCatch bag (medium or large, 15 mm) was introduced into the abdominal cavity. An endoscope with a snare was inserted orally and passed through the gastroduodenal surgical defect to grasp the bag, facilitating its extraction through the oral route (Fig. 1). Extraction was performed with careful, slow, and steady traction on the EndoCatch bag to minimize the risk of esophageal injury. Following specimen retrieval, an endoscope was reintroduced to inspect the esophagus, pharynx, and stomach for any potential mucosal injury, including subclinical mucosal tears or lacerations. After confirming the absence of injuries, the gastrotomy/duodenotomy was closed using 3-0 Vicryl/V-Loc absorbable sutures, reinforced with 3-0 PDS/silk sutures as necessary. The stomach was insufflated to confirm the absence of bleeding or leaks. An omental flap was placed over the sutured area to provide coverage and promote healing by providing a vascularized tissue layer. The abdominal cavity was irrigated with normal saline, and all residual fluid was aspirated. Hemostasis was confirmed, and the abdomen was deflated. Incisions were closed using Monocryl sutures. We prioritized safety throughout the procedures. In cases where complications such as significant bleeding, injury to adjacent organs, or inability to progress within a reasonable timeframe occurred, conversion to an open approach was prepared as a contingency plan.

### Data collection and outcome measures

Data on operative time, estimated blood loss (EBL), intraoperative events, postoperative pain, length of hospital stay, postoperative complications, diet advancement, pain scores, and pain management methods were collected through chart review using UCSF’s EPIC electronic medical record for each patient. Postoperative pain levels were assessed using a visual analog scale (0–10), with specific attention to comparing the highest pain level reported during hospitalization to the pain level recorded on the day of discharge. Diet advancement was tracked, noting when patients progressed from clear liquid to full-liquid diet. Postoperative follow-up was conducted to monitor for complications such as esophageal strictures, wound infections, or incisional hernias.

## Results

### Patient characteristics

**Fig. 2:**
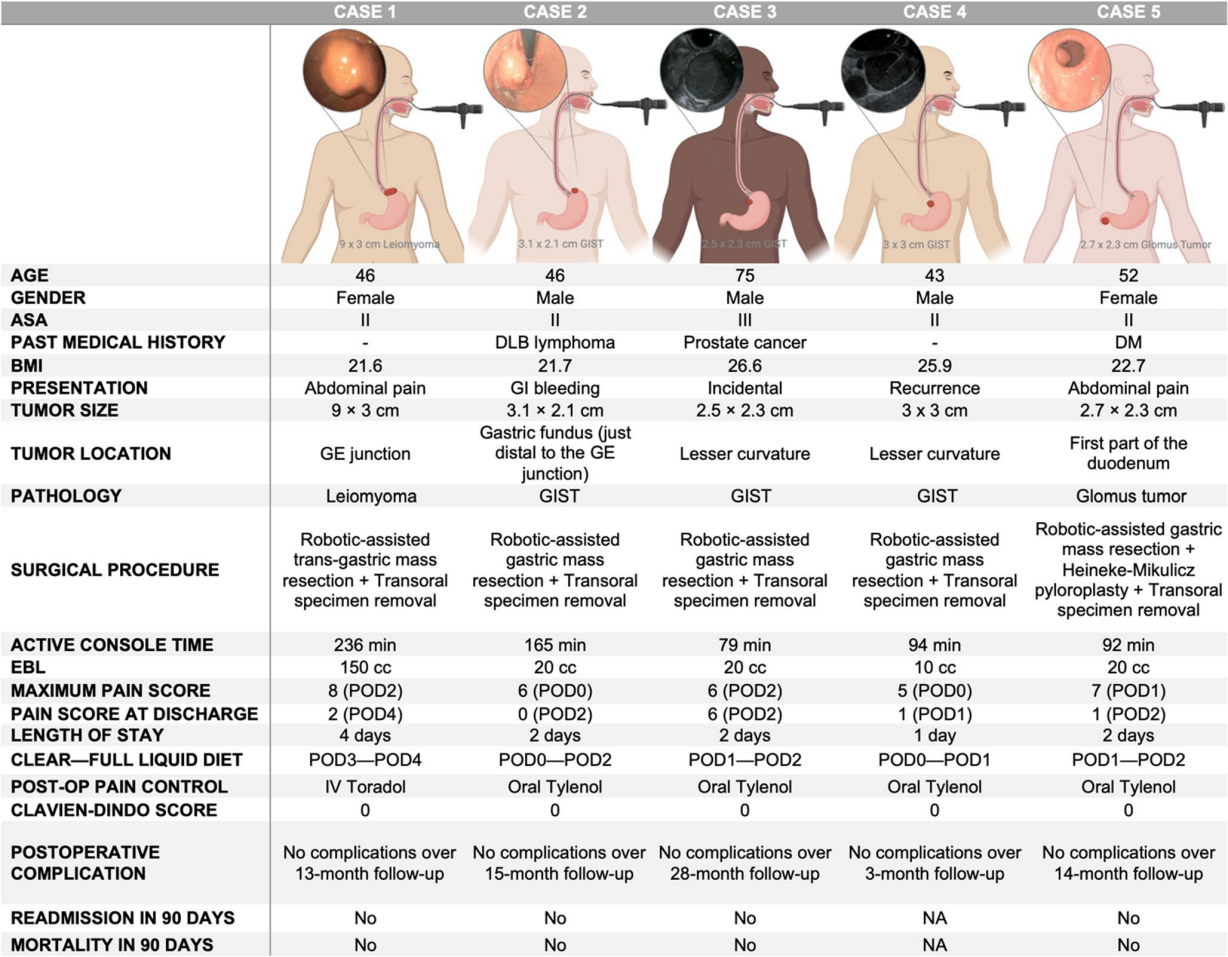
Demographics, tumor characteristics, and surgical outcomes for five patients undergoing transoral extraction following robotic-assisted resection of gastroduodenal tumors

The study included five patients with varying preoperative and pathologic characteristics (Fig. 2). The first patient was a 46-year-old female with a BMI of 21.6 kg/m² and ASA class II, who presented with abdominal pain and a history of benign gastric leiomyoma. Preoperative CT imaging revealed a 9.0 × 3.0 cm heterogeneous mass with mixed density and calcifications at the GEJ, which was confirmed by fine needle aspiration (FNA) as leiomyoma.

The second patient, a 46-year-old male with a BMI of 21.7 kg/m² and ASA class II, had a history of diffuse large B-cell lymphoma in remission and presented with gastrointestinal bleeding. CT scan showed a 3.1 × 2.1 cm gastric mass and significant mesenteric and retroperitoneal lymphadenopathy, with FNA identifying the mass as gastrointestinal stromal tumor (GIST).

The third patient was a 75-year-old male with a BMI of 26.6 kg/m² and ASA class III, with a history of prostate cancer and was incidentally found to have a 2.5 × 2.3 cm mass along the lesser curvature of the stomach, which was confirmed by FNA as GIST.

The fourth patient was a 43-year-old male with a BMI of 25.9 kg/m² and ASA class II, with no other medical history, presenting with a recurrence of a 3 × 3 cm GIST at the lesser curvature.

The fifth patient, a diabetic 52-year-old female with a BMI of 22.7 kg/m² and ASA class II, presented with abdominal pain. CT imaging demonstrated heterogeneously enhancing submucosal lesion in the gastric antrum, measuring 2.7 × 2.3 cm, without extension through the gastric wall; FNA identified the tumor as glomus tumor.

### Procedure

All patients underwent robotic-assisted tumor resection with successful transoral specimen extraction. The first patient had an intraluminal mass resection performed without causing injury or undue pressure on surrounding structures. The second, third, and fourth patients underwent robotic-assisted gastric mass wedge resections with transoral specimen extraction. The fifth patient had robotic-assisted wedge resection of a D1 duodenal mass, followed by Heineke-Mikulicz pyloroplasty. The median console time was 94 minutes (IQR 85.5–200.5), with an estimated blood loss (EBL) of 150 cc for the first patient and less than 20 cc for the remaining patients.

### Postoperative outcomes and follow-up

The first patient, who underwent resection for a gastroesophageal junction (GEJ) mass, started on a clear liquid diet on postoperative day (POD) #3 after passing a Gastrografin swallow study. This patient was advanced to full liquid diet by POD #4 and was subsequently discharged on the same day (POD #4).

The remaining four patients (80%) began clear liquid diet by POD #1 or earlier and advanced to full liquid diet by POD #2. The median length of hospital stay was two days, with 80% (4/5) of patients discharged on POD #2.

The median maximum pain score was 6, which decreased to a median of 1 at discharge. Pain management was achieved with oral Tylenol in 80% (4/5) of patients, while one patient required intravenous Toradol. All patients had effective pain control with non-narcotic medications by discharge.

No complications were reported in any patient, with a Clavien-Dindo classification of 0 for all cases. All patients were discharged in stable condition with scheduled follow-up appointments. Long-term follow-up demonstrated excellent outcomes, with no complications or readmissions reported at 13, 15, 28, 3, and 14 months of follow-up, respectively. No mortality was recorded for any of the cases. No incisional hernias were reported during the follow-up time.

## Discussion

To our knowledge, this study is the first robotic case series describing robotic-assisted resection of gastroduodenal tumors with structured transoral extraction. While transoral extraction has been described in the laparoscopic setting, its application within a robotic platform represents a technical evolution of established NOSES principles. The feasibility of transoral extraction is shown with its successful application across a range of tumor locations, from the gastroesophageal junction to the duodenum and for various non-adenocarcinoma pathologies, including leiomyomas, GISTs, and glomus tumors. The procedures were performed without intraoperative complications. Specimens were extracted with intact tumor retrieval. The median console time was 94 min, and no esophageal or oropharyngeal complications, readmissions, or mortality were observed throughout the follow-up period.

Transabdominal specimen extraction is the traditional approach in minimally invasive gastroduodenal surgeries, favored for its simplicity and surgeon familiarity. However, advancements in minimally invasive techniques, particularly NOSES, offer alternative options that eliminate the need for abdominal incisions [9]. Prior studies have shown that NOSES provides potential benefits over the traditional transabdominal approach, including reduced analgesic use, shorter hospital stays, less postoperative pain, faster oral intake resumption, fewer complications, and accelerated gastrointestinal recovery [10, 11].

The transvaginal route has been effective for extracting a variety of tumors, including gastric cancers, and is the most widely used technique in NOSES due to its ability to accommodate larger specimens [12, 13]. When performed properly, transvaginal extraction is not inherently associated with dyspareunia [14, 15]. However, transvaginal extraction is limited to female patients, and potential complications remain a consideration in patients with contraindications such as prior pelvic infections or strictures [16]. The transrectal route has also been explored in gastric surgeries and is occasionally used for extracting gastric specimens [17]. However, it is less frequently employed due to challenges such as the risk of sphincter injury, the potential for pelvic contamination, and complications like proctectomy leaks [18, 19]. Anatomical variations, such as anal stenosis or narrow rectal caliber, may further limit the feasibility of this approach [20].

The transoral route offers a unique alternative by enabling specimen extraction through the natural orifice of the mouth, thereby avoiding extra abdominal incisions and the need for extra-anatomic luminal openings [21–23]. This approach could offer distinct advantages, including reduced postoperative pain, the elimination of colonic or pelvic manipulation, and a lower risk of infections associated with additional luminal access compared to other techniques for gastric cancers [24, 25].

Preventing esophageal injury is a primary consideration for transoral extraction, especially when managing larger, irregularly shaped, or firm tumors. Ensuring the safety of this technique requires meticulous preoperative planning, intraoperative judgment, and adherence to strict guidelines. To minimize the risk of esophageal injury, preoperative imaging, and EGD were utilized for precise lesion measuring, excluding patients with esophageal varices or stenosis, to enhance procedural accuracy and safety. Current endoscopic retrieval guidelines recommend this approach for specimens with configurations of 2.5 cm in diameter and 6.0 cm in length [26]. In this case series, three patients had tumors approximately 3.0 cm in diameter, aligning with current practice, while one patient presented with a tumor measuring 9.0 cm in the maximal dimension but only 3.0 cm in the smallest dimension. This mass was oriented to utilize the smallest dimension for safe extraction through the esophagus, emphasizing the importance of considering specimen configuration and orientation when determining the feasibility and safety of transoral extraction.

During extraction, the use of a lubricated EndoCatch bag, and an endoscopic snare facilitated smooth and atraumatic retrieval. However, caution is paramount in such cases. To ensure patient safety remains the utmost priority, any resistance encountered during the extraction process should prompt immediate termination of the procedure. The specimen should then be pushed back into the stomach and the abdominal cavity for (traditional) transabdominal extraction. Additionally, oncologic safety and integrity should be upheld to mitigate the risk of tumor seeding during passage through the esophagus and mouth. The specimens were securely enclosed in EndoCatch bags, and careful handling techniques minimized compression, ensuring safe and effective retrieval. While no esophageal or oropharyngeal complications were observed, the sample size is insufficient to exclude rare but clinically significant adverse events.

Our findings underscore several key advantages of transoral extraction in robotic surgery for gastroduodenal tumors. By eliminating the need for skin incisions larger than 8 mm and avoiding additional abdominal incisions, transoral extraction may enhance immediate outcomes and lower the risk of incision-related complications, such as hernias and wound infections. The reduced tissue trauma minimizes postoperative pain and accelerates recovery. Patients in our study experienced short hospital stays (median of two days) and effective pain management with simple oral medications without the need for narcotics.

While the transoral approach demonstrates favorable outcomes, particularly for smaller benign gastroduodenal tumors, some limitations must be acknowledged. The narrow diameter and less elastic nature of the esophageal lumen present technical challenges, posing risks of esophageal rupture or bleeding during the passage of larger or bulkier specimens. Consequently, transoral extraction is currently most suitable for patients with smaller, benign tumors [27]. Careful patient selection remains essential to optimize safety and effectiveness, as ideal candidates are those with benign or low-grade gastric masses, without esophageal varices or stenosis, and no history of gastrointestinal bleeding or tumor-related perforation [28]. Patients with larger or bulkier tumors or those with conditions affecting esophageal elasticity may not be suitable candidates until further refinement of the technique allows for broader applicability.

The findings of the current study are limited by being conducted by a single surgeon at a single institution, which may affect the generalizability of the results. Furthermore, the strict exclusion of tumors exceeding 3.0 cm in the smallest dimension, while necessary for safety, defines a narrow patient selection window. For smaller specimens, the incremental benefit over minimal incision extension is likely modest and should be weighed against the added technical complexity. Also, it should be noted that postoperative endoscopic inspection, while reassuring, may not detect subclinical mucosal injury or intramural hematomas; future studies should incorporate functional imaging or manometric follow-up to characterize the esophageal safety of this technique more rigorously. Therefore, further research in larger, multicenter studies is essential to validate these findings across diverse patient populations and a broader range of tumor sizes and types. Comparative studies could also help define the advantages of transoral extraction relative to other NOSES techniques, such as transvaginal and transrectal approaches, particularly with respect to long-term outcomes and patient satisfaction.

Although the transoral extraction approach is still in its early stages of widespread adoption, the development of standardized protocols for robotic-assisted resection with transoral extraction could significantly enhance safety and consistency across different surgical settings. Future studies should focus on refining these protocols, expanding patient eligibility criteria, and optimizing factors such as patient selection, preoperative planning, surgical techniques, and postoperative monitoring to improve outcomes. In our series, all procedures were performed by a single surgeon with combined robotic and endoscopic expertise, demonstrating that a dual-operator setup is not strictly required. However, broader adoption may require structured training programs and interdisciplinary collaboration between robotic surgeons and advanced endoscopists, with standardized protocols to ensure reproducibility across centers [29]. As transoral extraction gains acceptance, surgeon proficiency and confidence in this technique will play a vital role in its successful integration into clinical practice. Ultimately, advancing transoral extraction through continued research, protocol refinement, and comprehensive training will help establish transoral extraction as a safe, effective, and cosmetically favorable option in minimally invasive gastroduodenal surgery.

## Conclusion

In conclusion, this case series demonstrates the technical feasibility of transoral specimen extraction following robotic resection of gastroduodenal tumors. In a carefully selected cohort, the technique can be associated with favorable short-term outcomes, including lower surgical morbidity and postoperative pain, with potentially reduced risk of complications such as wound infections or incisional hernia formation. By integrating the advantages of the robotic approach with the minimally invasive benefits of natural orifice specimen extraction, this technique may represent an advancement in the minimally invasive management of gastroduodenal tumors. However, further multicenter and comparative studies are required to establish its safety, reproducibility, and clinical value.

## Data Availability

No datasets were generated or analysed during the current study.
